# Orphenadrinium picrate

**DOI:** 10.1107/S1600536810049937

**Published:** 2010-12-18

**Authors:** Jerry P. Jasinski, Ray J. Butcher, B. P. Siddaraju, H. S. Yathirajan, B. Narayana

**Affiliations:** a Department of Chemistry, Keene State College, 229 Main Street, Keene, NH 03435-2001, USA; b Department of Chemistry, Howard University, 525 College Street NW, Washington, DC 20059, USA; c Department of Studies in Chemistry, University of Mysore, Manasagangotri, Mysore 570 006, India; d Department of Studies in Chemistry, Mangalore University, Mangalagangotri, 574 199, India

## Abstract

In the title molecular salt {systematic name: *N*,*N*-dimethyl-2-[(2-methyl­phen­yl)(phen­yl)meth­oxy]ethanaminium 2,4,6-tri­nitro­phenolate}, C_18_H_24_NO^+^·C_6_H_2_N_3_O_7_^−^, the phenyl rings of the orphenadrinum cation are disordered [occupancies = 0.662 (4) and 0.338 (4)]. The N atom in the orphenadrinum cation is protonated. The picrate anion inter­acts with the protonated N atom through a bifurcated N—H⋯O hydrogen bond, forming an *R*_1_^2^(6) ring motif with an adjacent cation. The mean planes of the two *o*-NO_2_ and single *p*-NO_2_ groups in the picrate anion are twisted by 23.0 (6), 31.3 (3) and 6.3 (2)° with respect to the mean planes of the six-membered ring. Weak inter­molecular C—H⋯O hydrogen bonds, C—H⋯π inter­molecular inter­actions and weak π–π stacking inter­actions [centroid–centroid distances = 3.677 (2) and 3.515 (3) Å} stabilize the crystal packing, creating a three-dimensional network.

## Related literature

For the pharmacological activity of the title compound, see: Hunskaar & Donnel (1991 ▶). For related structures, see: Fun *et al.* (2010 ▶); Glaser *et al.* (1992 ▶).
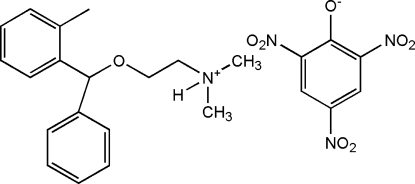


## Experimental

### 

#### Crystal data

C_18_H_24_NO^+^·C_6_H_2_N_3_O_7_^−^
*M* *_r_* = 498.49
Triclinic, 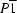

*a* = 9.9434 (10) Å
*b* = 11.2216 (8) Å
*c* = 11.3523 (12) Å
α = 78.658 (7)°
β = 76.342 (9)°
γ = 87.660 (7)°
*V* = 1206.82 (19) Å^3^
*Z* = 2
Cu *K*α radiation
μ = 0.88 mm^−1^
*T* = 123 K
0.52 × 0.43 × 0.16 mm

#### Data collection

Oxford Diffraction Xcalibur Ruby Gemini diffractometer
Absorption correction: multi-scan (*CrysAlis RED*; Oxford Diffraction, 2007 ▶) *T*_min_ = 0.635, *T*_max_ = 1.000
7402 measured reflections
4677 independent reflections
3760 reflections with *I* > 2σ(*I*)
*R*_int_ = 0.031

#### Refinement

*R*[*F* ^2^ > 2σ(*F* ^2^)] = 0.065
*wR*(*F* ^2^) = 0.188
*S* = 1.09
4677 reflections
407 parameters
H-atom parameters constrained
Δρ_max_ = 0.45 e Å^−3^
Δρ_min_ = −0.40 e Å^−3^

### 

Data collection: *CrysAlis PRO* (Oxford Diffraction, 2007 ▶); cell refinement: *CrysAlis PRO*; data reduction: *CrysAlis RED* (Oxford Diffraction, 2007 ▶); program(s) used to solve structure: *SHELXS97* (Sheldrick, 2008 ▶); program(s) used to refine structure: *SHELXL97* (Sheldrick, 2008 ▶); molecular graphics: *SHELXTL* (Sheldrick, 2008 ▶); software used to prepare material for publication: *SHELXTL*.

## Supplementary Material

Crystal structure: contains datablocks global, I. DOI: 10.1107/S1600536810049937/bt5424sup1.cif

Structure factors: contains datablocks I. DOI: 10.1107/S1600536810049937/bt5424Isup2.hkl

Additional supplementary materials:  crystallographic information; 3D view; checkCIF report

## Figures and Tables

**Table 1:** Hydrogen-bond geometry (Å, °) *Cg*2 and *Cg*3 are the centroids of the C9*A*–C7*A* and C2*C*–C7*C* rings, respectively.

*D*—H⋯*A*	*D*—H	H⋯*A*	*D*⋯*A*	*D*—H⋯*A*
N1*A*—H1*AB*⋯O1*B*	0.93	1.85	2.661 (2)	144
N1*A*—H1*AB*⋯O7*B*	0.93	2.36	3.031 (3)	129
C4*A*—H4*AA*⋯O4*B*^i^	0.95	2.46	3.346 (4)	155
C16*A*—H16*A*⋯O3*B*^ii^	0.99	2.57	3.519 (3)	160
C17*A*—H17*A*⋯O2*B*^ii^	0.98	2.57	3.470 (4)	153
C18*A*—H18*A*⋯O6*B*^iii^	0.98	2.41	3.167 (3)	133
C18*A*—H18*C*⋯O4*B*^iv^	0.98	2.36	3.317 (3)	166
C8*C*—H8*CB*⋯O6*B*	0.96	2.48	3.239 (9)	136
C6*A*—H6*AA*⋯*Cg*2^v^	0.93	2.88	3.643 (2)	138
C6*A*—H6*AA*⋯*Cg*3^v^	0.93	3.00	3.836 (4)	148
C12*C*—H12*B*⋯*Cg*2^v^	0.93	2.62	3.492 (4)	153
C12*C*—H12*B*⋯*Cg*3^v^	0.93	2.83	3.704 (4)	153

Symmetry codes: (i) 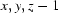; (ii) 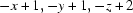; (iii) 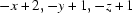; (iv) 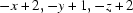; (v) 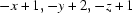.

**Fig. 1.**
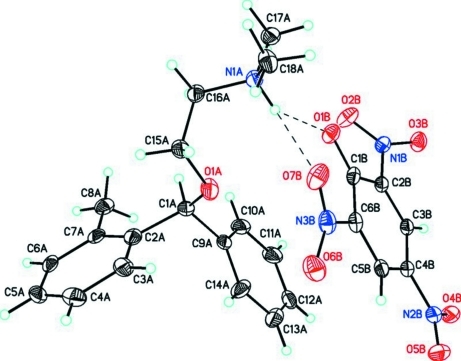
Molecular structure of the title compound, (I), showing the atom labeling scheme and 50% probability displacement ellipsoids. Only the highest occupied atoms in the disordered phenyl rings [occupancy C1A–C14A = 0.662 (4)] are shown. Dashed lines indicate weak C—H···O intermolecular hydrogen bonds between the cation and anion and R12(6) ring motifs.

**Fig. 2.**
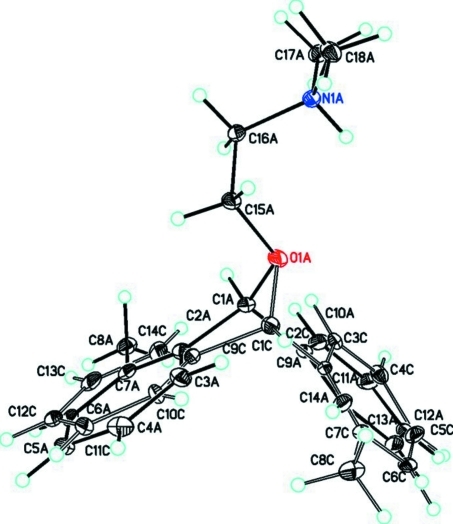
Molecular structure of the title compound, (I), showing the disordered atoms in the two phenyl rings [occupancy C1A–C14A = 0.662 (4); C1C–C13C = 0.338 (4)] of the orphenadrinium cation.

**Fig. 3.**
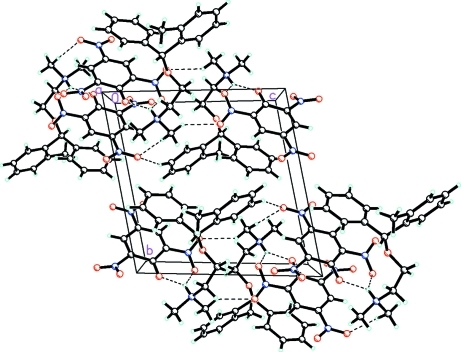
Packing diagram of the title compound viewed down the a axis. Only the highest occupied atoms in the disordered phenyl rings [occupancy C1A–C14A = 0.662 (4)] are shown. Dashed lines indicate weak intermolecular C—H···O hydrogen bond interactions creating a 3-D supramolecular structure.

## supplementary crystallographic information

### Comment

Orphenadrine (systematic IUPAC name: N, *N*-dimethyl-2-[(2-methylphenyl) phenyl-methoxy]ethanamine) is an anticholinergic drug of the ethanolamine antihistamine class with prominent *CNS* and peripheral actions used to treat painful muscle spasm and other symptoms and conditions as well as some aspects of Parkinson's disease. It is closely related to diphenhydramine and therefore related to other drugs used for Parkinson's like benztropine and trihexyphenidyl and is also structurally related to nefopam, a centrally acting yet non-opioid analgesic. Clinical and pharmacological review of the efficacy of orphenadrine and its combination with paracetamol has been described (Hunskaar & Donnel, 1991).

The solid-state structure of orphenadrine hydrochloride and conformational comparisons with diphenhydramine hydrochloride and nefopam hydrochloride was reported (Glaser *et al.*, 1992). The crystal structure of orphenadrinium picrate picric acid is recently reported (Fun *et al.*, 2010). The present work reports the crystal structure of the title compound, (I), which was obtained by the interaction between orphenadrine hydrochloride and 2,4,6-trinitrophenol in aqueous medium.

In the crystal structure of the title compound, C_18_H_24_NO^+^. C_6_H_2_N_3_O_7_^-^, there is one cation-anion pair in the asymmetric unit (Fig. 1). The two phenyl rings in the orphenadrinum cation are disordered [occupancy C1A–C14A = 0.662 (4); C1C–C13C = 0.338 (4)] with a protonated N atom in the *N*-dimethylethanamine group (Fig. 2). The dihedral angle between the mean planes of the two cation phenyl rings [occupancy C1A–C14A = 0.662 (4)] is 73.2 (1)°. The picrate anion interacts with the protonated N atom through a bifurcated N—H···O hydrogen bond forming a *R*_1_^2^(6) ring motif with an adjacent cation. The dihedral angle between the mean planes of the anion benzene and two cation phenyl rings [occupancy C1A–C14A = 0.662 (4)] is 77.2 (6)° and 9.7 (0)°, respectively. The mean planes of the two *o*-NO_2_ and single *p*-NO_2_ groups in the picrate anion are twisted by 23.0 (6)°, 31.3 (3)° and 6.3 (2)° with respect to the mean planes of the 6-membered benzene ring. Weak Intermolecular C—H···O hydrogen bonds, C—H···*Cg* intermolecular interactions (Table 1), and weak π–π stacking interactions (Table 2) dominate the crystal packing creating a 3-D supramolecular structure (Fig. 3).

### Experimental

Orphenadrine hydrochloride (3.05 g, 0.01 mol) was dissolved in 25 ml of water and picric acid (2.4 g, 0.01 mol) was also dissolved in 25 ml of water. Both solutions were mixed and stirred in a beaker at room temperature for 1 h. The mixture was warmed at 323 K for 10 min & kept aside for 2 days at room temperature. The formed product was filtered and dried in vaccum desiccator over phosphorous pentoxide. The product was recrystallized from dimethyl sulphoxide by slow evaporation (m.p. 341–344 K).

### Refinement

The two o-phenyl rings in the orphenadrinium cation are disordered [occupancy C1A–C14A = 0.662 (4); C1C–C13C = 0.338 (4)]. All of the H atoms were placed in their calculated positions and then refined using the riding model with Atom—H lengths of 0.95Å (CH), 0.96 & 0.99Å (CH_2_), 0.98Å (CH_3_) or 0.93Å (NH). Isotropic displacement parameters for these atoms were set to 1.19 times (NH), 1.19–1.21 (CH, CH_2_) or 1.49–1.50 (CH_3_) times *U*_eq_ of the parent atom.

### Crystal data

**Table:**

C_18_H_24_NO^+^·C_6_H_2_N_3_O_7_^−^	*Z* = 2
*M**_r_* = 498.49	*F*(000) = 524
Triclinic, *P*1	*D*_x_ = 1.372 Mg m^−^^3^
Hall symbol: -P 1	Cu *K*α radiation, λ = 1.54184 Å
*a* = 9.9434 (10) Å	Cell parameters from 3744 reflections
*b* = 11.2216 (8) Å	θ = 4.6–74.4°
*c* = 11.3523 (12) Å	µ = 0.88 mm^−^^1^
α = 78.658 (7)°	*T* = 123 K
β = 76.342 (9)°	Triangular plate, yellow
γ = 87.660 (7)°	0.52 × 0.43 × 0.16 mm
*V* = 1206.82 (19) Å^3^	

### Data collection

**Table:**

Oxford Diffraction Xcalibur Ruby Gemini diffractometer	4677 independent reflections
Radiation source: Enhance (Cu) X-ray Source	3760 reflections with *I* > 2σ(*I*)
graphite	*R*_int_ = 0.031
Detector resolution: 10.5081 pixels mm^-1^	θ_max_ = 74.6°, θ_min_ = 4.6°
ω scans	*h* = −12→12
Absorption correction: multi-scan (*CrysAlis RED*; Oxford Diffraction, 2007)	*k* = −13→13
*T*_min_ = 0.635, *T*_max_ = 1.000	*l* = −8→14
7402 measured reflections	

### Refinement

**Table:**

Refinement on *F*^2^	Secondary atom site location: difference Fourier map
Least-squares matrix: full	Hydrogen site location: inferred from neighbouring sites
*R*[*F*^2^ > 2σ(*F*^2^)] = 0.065	H-atom parameters constrained
*wR*(*F*^2^) = 0.188	*w* = 1/[σ^2^(*F*_o_^2^) + (0.093*P*)^2^ + 0.5659*P*] where *P* = (*F*_o_^2^ + 2*F*_c_^2^)/3
*S* = 1.09	(Δ/σ)_max_ < 0.001
4677 reflections	Δρ_max_ = 0.45 e Å^−^^3^
407 parameters	Δρ_min_ = −0.40 e Å^−^^3^
0 restraints	Extinction correction: *SHELXL97* (Sheldrick, 2008), Fc^*^=kFc[1+0.001xFc^2^λ^3^/sin(2θ)]^-1/4^
Primary atom site location: structure-invariant direct methods	Extinction coefficient: 0.0038 (11)

### Special details

**Table:**

Geometry. All e.s.d.'s (except the e.s.d. in the dihedral angle between two l.s. planes) are estimated using the full covariance matrix. The cell e.s.d.'s are taken into account individually in the estimation of e.s.d.'s in distances, angles and torsion angles; correlations between e.s.d.'s in cell parameters are only used when they are defined by crystal symmetry. An approximate (isotropic) treatment of cell e.s.d.'s is used for estimating e.s.d.'s involving l.s. planes.
Refinement. Refinement of *F*^2^ against ALL reflections. The weighted *R*-factor *wR* and goodness of fit *S* are based on *F*^2^, conventional *R*-factors *R* are based on *F*, with *F* set to zero for negative *F*^2^. The threshold expression of *F*^2^ > σ(*F*^2^) is used only for calculating *R*-factors(gt) *etc*. and is not relevant to the choice of reflections for refinement. *R*-factors based on *F*^2^ are statistically about twice as large as those based on *F*, and *R*- factors based on ALL data will be even larger.

### Fractional atomic coordinates and isotropic or equivalent isotropic displacement parameters (Å^2^)

**Table:**

	*x*	*y*	*z*	*U*_iso_*/*U*_eq_	Occ. (<1)
O1A	0.7336 (3)	0.61869 (16)	0.6143 (2)	0.0829 (7)	
N1A	0.7148 (2)	0.35671 (16)	0.72631 (17)	0.0460 (5)	
H1AB	0.7569	0.4148	0.7541	0.055*	
C1A	0.6397 (4)	0.7080 (3)	0.6259 (3)	0.0453 (9)	0.662 (4)
H1AA	0.5486	0.6683	0.6695	0.054*	0.662 (4)
C2A	0.6208 (5)	0.7849 (4)	0.5018 (4)	0.051 (2)	0.662 (4)
C3A	0.7346 (4)	0.8060 (4)	0.4020 (6)	0.0592 (16)	0.662 (4)
H3AA	0.8210	0.7696	0.4090	0.071*	0.662 (4)
C4A	0.7220 (4)	0.8805 (3)	0.2918 (4)	0.0681 (13)	0.662 (4)
H4AA	0.7998	0.8949	0.2236	0.082*	0.662 (4)
C5A	0.5956 (6)	0.9339 (3)	0.2815 (3)	0.0590 (14)	0.662 (4)
H5AA	0.5870	0.9848	0.2062	0.071*	0.662 (4)
C6A	0.4818 (4)	0.9128 (4)	0.3813 (5)	0.0531 (17)	0.662 (4)
H6AA	0.3954	0.9493	0.3743	0.064*	0.662 (4)
C7A	0.4944 (4)	0.8383 (3)	0.4915 (4)	0.0464 (11)	0.662 (4)
C8A	0.3672 (4)	0.8167 (3)	0.5954 (4)	0.0581 (11)	0.662 (4)
H8AA	0.2909	0.8607	0.5698	0.087*	0.662 (4)
H8AB	0.3454	0.7315	0.6177	0.087*	0.662 (4)
H8AC	0.3842	0.8445	0.6654	0.087*	0.662 (4)
C9A	0.6762 (5)	0.7860 (3)	0.7096 (4)	0.0536 (11)	0.662 (4)
C10A	0.5891 (5)	0.7873 (3)	0.8248 (4)	0.0708 (15)	0.662 (4)
H10A	0.5069	0.7396	0.8512	0.085*	0.662 (4)
C11A	0.6224 (6)	0.8585 (4)	0.9013 (3)	0.087 (2)	0.662 (4)
H11A	0.5629	0.8594	0.9801	0.104*	0.662 (4)
C12A	0.7428 (6)	0.9283 (4)	0.8627 (4)	0.075 (3)	0.662 (4)
H12A	0.7656	0.9769	0.9150	0.091*	0.662 (4)
C13A	0.8299 (5)	0.9269 (4)	0.7475 (5)	0.081 (2)	0.662 (4)
H13A	0.9121	0.9747	0.7210	0.097*	0.662 (4)
C14A	0.7966 (4)	0.8558 (4)	0.6709 (4)	0.0721 (18)	0.662 (4)
H14A	0.8561	0.8549	0.5922	0.087*	0.662 (4)
C15A	0.7104 (3)	0.5276 (2)	0.5497 (2)	0.0519 (6)	
H15A	0.8002	0.4997	0.5049	0.062*	
H15B	0.6562	0.5621	0.4884	0.062*	
C16A	0.6334 (3)	0.4220 (2)	0.6388 (2)	0.0473 (5)	
H16A	0.5464	0.4517	0.6867	0.057*	
H16B	0.6085	0.3642	0.5917	0.057*	
C17A	0.6223 (3)	0.2806 (2)	0.8360 (2)	0.0589 (7)	
H17A	0.5585	0.3332	0.8822	0.088*	
H17B	0.6785	0.2345	0.8895	0.088*	
H17C	0.5694	0.2241	0.8084	0.088*	
C18A	0.8262 (3)	0.2799 (2)	0.6658 (2)	0.0598 (7)	
H18A	0.8846	0.3300	0.5925	0.090*	
H18B	0.7841	0.2140	0.6414	0.090*	
H18C	0.8828	0.2454	0.7240	0.090*	
C1C	0.7475 (7)	0.7270 (6)	0.5766 (6)	0.0435 (16)	0.338 (4)
H1CA	0.8407	0.7363	0.5182	0.052*	0.338 (4)
C2C	0.7572 (7)	0.8018 (5)	0.6752 (4)	0.0309 (14)	0.338 (4)
C3C	0.6651 (6)	0.7736 (5)	0.7898 (6)	0.0416 (17)	0.338 (4)
H3CA	0.5985	0.7106	0.8053	0.050*	0.338 (4)
C4C	0.6704 (8)	0.8374 (8)	0.8819 (5)	0.053 (2)	0.338 (4)
H4CA	0.6074	0.8181	0.9603	0.064*	0.338 (4)
C5C	0.7678 (11)	0.9295 (8)	0.8593 (7)	0.075 (5)	0.338 (4)
H5CA	0.7714	0.9731	0.9223	0.090*	0.338 (4)
C6C	0.8599 (9)	0.9577 (7)	0.7447 (8)	0.057 (3)	0.338 (4)
H6CA	0.9265	1.0207	0.7293	0.068*	0.338 (4)
C7C	0.8547 (6)	0.8939 (6)	0.6526 (5)	0.0482 (19)	0.338 (4)
C8C	0.9529 (9)	0.9303 (7)	0.5317 (9)	0.067 (2)	0.338 (4)
H8CA	1.0122	0.9947	0.5342	0.101*	0.338 (4)
H8CB	1.0081	0.8620	0.5104	0.101*	0.338 (4)
H8CC	0.9002	0.9581	0.4708	0.101*	0.338 (4)
C9C	0.6485 (10)	0.7893 (8)	0.4994 (8)	0.045 (3)	0.338 (4)
C10C	0.7073 (8)	0.8359 (8)	0.3758 (8)	0.057 (3)	0.338 (4)
H10B	0.8034	0.8260	0.3434	0.069*	0.338 (4)
C11C	0.6255 (12)	0.8969 (7)	0.2996 (7)	0.081 (5)	0.338 (4)
H11B	0.6656	0.9287	0.2152	0.097*	0.338 (4)
C12C	0.4848 (11)	0.9113 (8)	0.3471 (10)	0.064 (4)	0.338 (4)
H12B	0.4289	0.9530	0.2950	0.077*	0.338 (4)
C13C	0.4260 (8)	0.8648 (8)	0.4707 (10)	0.074 (3)	0.338 (4)
H13B	0.3299	0.8746	0.5031	0.089*	0.338 (4)
C14C	0.5079 (11)	0.8038 (7)	0.5468 (7)	0.069 (3)	0.338 (4)
H14B	0.4677	0.7720	0.6313	0.083*	0.338 (4)
O1B	0.78560 (18)	0.46162 (16)	0.89387 (15)	0.0545 (4)	
O2B	0.6057 (2)	0.4845 (2)	1.10418 (19)	0.0708 (6)	
O3B	0.71427 (19)	0.50961 (19)	1.23996 (16)	0.0624 (5)	
O4B	1.0321 (2)	0.85253 (18)	1.1050 (2)	0.0723 (6)	
O5B	1.1774 (2)	0.8672 (2)	0.9274 (2)	0.0818 (7)	
O6B	1.0764 (2)	0.6666 (2)	0.62610 (18)	0.0766 (6)	
O7B	0.98789 (19)	0.4863 (2)	0.68726 (18)	0.0680 (6)	
N1B	0.70630 (19)	0.52022 (17)	1.13249 (17)	0.0461 (5)	
N2B	1.0733 (3)	0.8227 (2)	1.0032 (2)	0.0606 (6)	
N3B	1.0114 (2)	0.5869 (2)	0.70603 (19)	0.0574 (6)	
C1B	0.8494 (2)	0.5445 (2)	0.9157 (2)	0.0429 (5)	
C2B	0.8186 (2)	0.58148 (19)	1.03509 (19)	0.0406 (5)	
C3B	0.8897 (2)	0.6690 (2)	1.0645 (2)	0.0434 (5)	
H3BA	0.8658	0.6876	1.1449	0.052*	
C4B	0.9972 (2)	0.7300 (2)	0.9748 (2)	0.0474 (5)	
C5B	1.0340 (2)	0.7033 (2)	0.8576 (2)	0.0491 (6)	
H5BA	1.1069	0.7469	0.7967	0.059*	
C6B	0.9642 (2)	0.6133 (2)	0.8304 (2)	0.0466 (5)	

### Atomic displacement parameters (Å^2^)

**Table:**

	*U*^11^	*U*^22^	*U*^33^	*U*^12^	*U*^13^	*U*^23^
O1A	0.140 (2)	0.0331 (9)	0.1071 (17)	0.0224 (10)	−0.0862 (16)	−0.0234 (10)
N1A	0.0622 (12)	0.0382 (9)	0.0454 (10)	0.0079 (8)	−0.0212 (9)	−0.0178 (8)
C1A	0.057 (2)	0.0313 (15)	0.0513 (19)	−0.0021 (14)	−0.0189 (17)	−0.0076 (14)
C2A	0.062 (4)	0.031 (3)	0.071 (5)	0.005 (2)	−0.030 (3)	−0.016 (3)
C3A	0.058 (3)	0.041 (3)	0.079 (4)	0.008 (2)	−0.026 (3)	−0.004 (3)
C4A	0.080 (3)	0.049 (3)	0.070 (3)	−0.006 (2)	−0.008 (3)	−0.008 (2)
C5A	0.091 (4)	0.035 (3)	0.054 (3)	−0.005 (2)	−0.029 (3)	−0.0009 (19)
C6A	0.073 (4)	0.032 (3)	0.072 (4)	0.004 (2)	−0.044 (3)	−0.020 (3)
C7A	0.055 (3)	0.036 (2)	0.056 (3)	−0.0089 (19)	−0.018 (2)	−0.019 (2)
C8A	0.057 (2)	0.046 (2)	0.077 (3)	0.0000 (16)	−0.018 (2)	−0.0214 (19)
C9A	0.082 (4)	0.0247 (16)	0.065 (3)	−0.0023 (19)	−0.044 (3)	−0.0008 (18)
C10A	0.111 (5)	0.043 (2)	0.064 (3)	−0.020 (3)	−0.033 (3)	−0.005 (2)
C11A	0.160 (7)	0.047 (3)	0.064 (3)	−0.026 (4)	−0.050 (4)	−0.003 (2)
C12A	0.140 (6)	0.035 (4)	0.073 (5)	0.000 (4)	−0.073 (4)	−0.003 (3)
C13A	0.093 (4)	0.031 (3)	0.145 (7)	0.011 (3)	−0.079 (4)	−0.021 (3)
C14A	0.074 (4)	0.041 (3)	0.121 (5)	0.001 (2)	−0.054 (4)	−0.025 (3)
C15A	0.0719 (16)	0.0363 (11)	0.0548 (13)	0.0091 (10)	−0.0261 (12)	−0.0140 (10)
C16A	0.0583 (13)	0.0382 (11)	0.0546 (13)	0.0084 (9)	−0.0244 (11)	−0.0189 (10)
C17A	0.0827 (18)	0.0481 (13)	0.0508 (14)	−0.0061 (12)	−0.0209 (13)	−0.0134 (11)
C18A	0.0833 (18)	0.0522 (14)	0.0529 (14)	0.0280 (13)	−0.0283 (13)	−0.0222 (11)
C1C	0.048 (4)	0.041 (3)	0.041 (3)	0.000 (3)	−0.010 (3)	−0.008 (3)
C2C	0.030 (3)	0.027 (4)	0.036 (3)	−0.005 (3)	−0.011 (3)	−0.001 (3)
C3C	0.049 (4)	0.040 (4)	0.037 (4)	0.006 (3)	−0.019 (4)	0.001 (3)
C4C	0.067 (5)	0.049 (5)	0.048 (4)	0.021 (4)	−0.021 (4)	−0.017 (4)
C5C	0.076 (7)	0.036 (8)	0.133 (16)	0.004 (5)	−0.057 (9)	−0.029 (9)
C6C	0.097 (7)	0.021 (4)	0.067 (6)	0.004 (4)	−0.045 (5)	−0.014 (3)
C7C	0.058 (5)	0.029 (4)	0.063 (5)	−0.001 (3)	−0.027 (4)	−0.007 (3)
C8C	0.066 (5)	0.050 (4)	0.080 (6)	−0.009 (4)	−0.006 (4)	−0.010 (4)
C9C	0.070 (7)	0.029 (5)	0.047 (7)	0.001 (4)	−0.032 (6)	−0.012 (4)
C10C	0.072 (6)	0.043 (6)	0.066 (7)	0.005 (4)	−0.045 (6)	0.001 (5)
C11C	0.138 (14)	0.036 (6)	0.092 (9)	−0.004 (7)	−0.079 (10)	−0.001 (5)
C12C	0.102 (11)	0.029 (5)	0.083 (8)	0.002 (5)	−0.056 (8)	−0.019 (5)
C13C	0.096 (8)	0.050 (5)	0.100 (10)	−0.004 (6)	−0.047 (8)	−0.038 (6)
C14C	0.110 (10)	0.048 (6)	0.075 (7)	0.018 (5)	−0.054 (7)	−0.039 (5)
O1B	0.0585 (10)	0.0651 (11)	0.0472 (9)	−0.0057 (8)	−0.0170 (7)	−0.0212 (8)
O2B	0.0593 (11)	0.0916 (15)	0.0678 (12)	−0.0224 (10)	−0.0065 (9)	−0.0355 (11)
O3B	0.0582 (10)	0.0834 (13)	0.0450 (9)	−0.0066 (9)	−0.0144 (8)	−0.0062 (9)
O4B	0.0985 (15)	0.0594 (11)	0.0697 (13)	−0.0230 (10)	−0.0421 (11)	−0.0059 (10)
O5B	0.0861 (15)	0.0765 (14)	0.0795 (14)	−0.0389 (12)	−0.0260 (12)	0.0090 (11)
O6B	0.0816 (14)	0.0921 (16)	0.0471 (11)	0.0039 (12)	−0.0065 (10)	−0.0027 (10)
O7B	0.0557 (11)	0.0980 (16)	0.0585 (11)	0.0003 (10)	−0.0114 (8)	−0.0363 (11)
N1B	0.0460 (10)	0.0497 (11)	0.0460 (10)	−0.0003 (8)	−0.0126 (8)	−0.0148 (8)
N2B	0.0753 (15)	0.0520 (12)	0.0589 (13)	−0.0151 (11)	−0.0351 (12)	0.0054 (10)
N3B	0.0517 (11)	0.0799 (16)	0.0392 (11)	0.0083 (10)	−0.0123 (9)	−0.0079 (11)
C1B	0.0445 (11)	0.0504 (12)	0.0400 (11)	0.0042 (9)	−0.0205 (9)	−0.0115 (9)
C2B	0.0436 (11)	0.0416 (11)	0.0403 (11)	0.0020 (8)	−0.0177 (9)	−0.0069 (9)
C3B	0.0520 (12)	0.0425 (11)	0.0412 (11)	0.0013 (9)	−0.0236 (10)	−0.0056 (9)
C4B	0.0529 (13)	0.0437 (11)	0.0501 (13)	−0.0050 (9)	−0.0282 (10)	0.0009 (10)
C5B	0.0473 (12)	0.0542 (13)	0.0440 (12)	−0.0009 (10)	−0.0195 (10)	0.0059 (10)
C6B	0.0460 (12)	0.0581 (13)	0.0390 (11)	0.0088 (10)	−0.0190 (9)	−0.0078 (10)

### Geometric parameters (Å, °)

**Table:**

O1A—C1C	1.208 (7)	C1C—C2C	1.548 (8)
O1A—C1A	1.346 (4)	C1C—H1CA	1.0000
O1A—C15A	1.425 (3)	C2C—C3C	1.3900
N1A—C16A	1.491 (3)	C2C—C7C	1.3900
N1A—C17A	1.494 (3)	C3C—C4C	1.3900
N1A—C18A	1.495 (3)	C3C—H3CA	0.9500
N1A—H1AB	0.9300	C4C—C5C	1.3900
C1A—C9A	1.522 (4)	C4C—H4CA	0.9500
C1A—C2A	1.549 (5)	C5C—C6C	1.3900
C1A—H1AA	1.0000	C5C—H5CA	0.9500
C2A—C3A	1.3900	C6C—C7C	1.3900
C2A—C7A	1.3900	C6C—H6CA	0.9500
C3A—C4A	1.3900	C7C—C8C	1.478 (10)
C3A—H3AA	0.9500	C8C—C8C^i^	1.789 (15)
C4A—C5A	1.3900	C8C—H8CA	0.9600
C4A—H4AA	0.9500	C8C—H8CB	0.9600
C5A—C6A	1.3900	C8C—H8CC	0.9601
C5A—H5AA	0.9500	C9C—C10C	1.3900
C6A—C7A	1.3900	C9C—C14C	1.3900
C6A—H6AA	0.9500	C10C—C11C	1.3900
C7A—C8A	1.503 (6)	C10C—H10B	0.9500
C8A—H8AA	0.9600	C11C—C12C	1.3900
C8A—H8AB	0.9600	C11C—H11B	0.9500
C8A—H8AC	0.9601	C12C—C13C	1.3900
C9A—C10A	1.3900	C12C—H12B	0.9500
C9A—C14A	1.3900	C13C—C14C	1.3900
C10A—C11A	1.3900	C13C—H8AA	1.5316
C10A—H10A	0.9500	C13C—H13B	0.9500
C11A—C12A	1.3900	C14C—H14B	0.9500
C11A—H11A	0.9500	O1B—C1B	1.242 (3)
C12A—C13A	1.3900	O2B—N1B	1.225 (3)
C12A—H12A	0.9500	O3B—N1B	1.223 (3)
C13A—C14A	1.3900	O4B—N2B	1.238 (3)
C13A—H13A	0.9500	O5B—N2B	1.231 (3)
C14A—H14A	0.9500	O6B—N3B	1.221 (3)
C15A—C16A	1.501 (3)	O7B—N3B	1.230 (3)
C15A—H15A	0.9900	N1B—C2B	1.457 (3)
C15A—H15B	0.9900	N2B—C4B	1.438 (3)
C16A—H16A	0.9900	N3B—C6B	1.463 (3)
C16A—H16B	0.9900	C1B—C6B	1.447 (3)
C17A—H17A	0.9800	C1B—C2B	1.455 (3)
C17A—H17B	0.9800	C2B—C3B	1.369 (3)
C17A—H17C	0.9800	C3B—C4B	1.385 (3)
C18A—H18A	0.9800	C3B—H3BA	0.9500
C18A—H18B	0.9800	C4B—C5B	1.383 (3)
C18A—H18C	0.9800	C5B—C6B	1.371 (3)
C1C—C9C	1.528 (8)	C5B—H5BA	0.9500
			
C1C—O1A—C1A	50.3 (4)	O1A—C1C—C9C	117.4 (6)
C1C—O1A—C15A	128.2 (4)	O1A—C1C—C2C	114.9 (5)
C1A—O1A—C15A	117.7 (2)	C9C—C1C—C2C	109.5 (6)
C16A—N1A—C17A	110.9 (2)	O1A—C1C—H1CA	104.5
C16A—N1A—C18A	112.23 (17)	C9C—C1C—H1CA	104.5
C17A—N1A—C18A	110.01 (19)	C2C—C1C—H1CA	104.5
C16A—N1A—H1AB	107.9	C3C—C2C—C7C	120.0
C17A—N1A—H1AB	107.9	C3C—C2C—C1C	117.8 (5)
C18A—N1A—H1AB	107.9	C7C—C2C—C1C	122.2 (5)
O1A—C1A—C9A	108.7 (3)	C2C—C3C—C4C	120.0
O1A—C1A—C2A	114.5 (3)	C2C—C3C—H3CA	120.0
C9A—C1A—C2A	112.2 (3)	C4C—C3C—H3CA	120.0
O1A—C1A—H1AA	107.0	C3C—C4C—C5C	120.0
C9A—C1A—H1AA	107.0	C3C—C4C—H4CA	120.0
C2A—C1A—H1AA	107.0	C5C—C4C—H4CA	120.0
C3A—C2A—C7A	120.0	C4C—C5C—C6C	120.0
C3A—C2A—C1A	118.9 (4)	C4C—C5C—H5CA	120.0
C7A—C2A—C1A	121.0 (4)	C6C—C5C—H5CA	120.0
C4A—C3A—C2A	120.0	C5C—C6C—C7C	120.0
C4A—C3A—H3AA	120.0	C5C—C6C—H6CA	120.0
C2A—C3A—H3AA	120.0	C7C—C6C—H6CA	120.0
C3A—C4A—C5A	120.0	C6C—C7C—C2C	120.0
C3A—C4A—H4AA	120.0	C6C—C7C—C8C	117.5 (6)
C5A—C4A—H4AA	120.0	C2C—C7C—C8C	122.5 (6)
C4A—C5A—C6A	120.0	C7C—C8C—C8C^i^	129.1 (9)
C4A—C5A—H5AA	120.0	C7C—C8C—H8CA	110.3
C6A—C5A—H5AA	120.0	C7C—C8C—H8CB	110.1
C7A—C6A—C5A	120.0	C8C^i^—C8C—H8CB	111.1
C7A—C6A—H6AA	120.0	H8CA—C8C—H8CB	109.5
C5A—C6A—H6AA	120.0	C7C—C8C—H8CC	108.0
C6A—C7A—C2A	120.0	C8C^i^—C8C—H8CC	84.9
C6A—C7A—C8A	117.6 (4)	H8CA—C8C—H8CC	109.5
C2A—C7A—C8A	122.3 (4)	H8CB—C8C—H8CC	109.5
C7A—C8A—H8AA	109.6	C10C—C9C—C14C	120.0
C7A—C8A—H8AB	109.7	C10C—C9C—C1C	116.2 (7)
H8AA—C8A—H8AB	109.5	C14C—C9C—C1C	123.8 (7)
C7A—C8A—H8AC	109.2	C11C—C10C—C9C	120.0
H8AA—C8A—H8AC	109.5	C11C—C10C—H10B	120.0
H8AB—C8A—H8AC	109.5	C9C—C10C—H10B	120.0
C10A—C9A—C14A	120.0	C10C—C11C—C12C	120.0
C10A—C9A—C1A	120.0 (3)	C10C—C11C—H11B	120.0
C14A—C9A—C1A	120.0 (3)	C12C—C11C—H11B	120.0
C9A—C10A—C11A	120.0	C11C—C12C—C13C	120.0
C9A—C10A—H10A	120.0	C11C—C12C—H12B	120.0
C11A—C10A—H10A	120.0	C13C—C12C—H12B	120.0
C12A—C11A—C10A	120.0	C14C—C13C—C12C	120.0
C12A—C11A—H11A	120.0	C14C—C13C—H8AA	97.3
C10A—C11A—H11A	120.0	C12C—C13C—H8AA	142.5
C11A—C12A—C13A	120.0	C14C—C13C—H13B	120.0
C11A—C12A—H12A	120.0	C12C—C13C—H13B	120.0
C13A—C12A—H12A	120.0	C13C—C14C—C9C	120.0
C14A—C13A—C12A	120.0	C13C—C14C—H14B	120.0
C14A—C13A—H13A	120.0	C9C—C14C—H14B	120.0
C12A—C13A—H13A	120.0	O3B—N1B—O2B	122.4 (2)
C13A—C14A—C9A	120.0	O3B—N1B—C2B	118.62 (18)
C13A—C14A—H14A	120.0	O2B—N1B—C2B	118.94 (19)
C9A—C14A—H14A	120.0	O5B—N2B—O4B	122.7 (2)
O1A—C15A—C16A	110.1 (2)	O5B—N2B—C4B	119.3 (2)
O1A—C15A—H15A	109.6	O4B—N2B—C4B	118.0 (2)
C16A—C15A—H15A	109.6	O6B—N3B—O7B	123.2 (2)
O1A—C15A—H15B	109.6	O6B—N3B—C6B	117.2 (2)
C16A—C15A—H15B	109.6	O7B—N3B—C6B	119.5 (2)
H15A—C15A—H15B	108.2	O1B—C1B—C6B	125.6 (2)
N1A—C16A—C15A	113.0 (2)	O1B—C1B—C2B	122.9 (2)
N1A—C16A—H16A	109.0	C6B—C1B—C2B	111.48 (19)
C15A—C16A—H16A	109.0	C3B—C2B—C1B	124.7 (2)
N1A—C16A—H16B	109.0	C3B—C2B—N1B	116.68 (19)
C15A—C16A—H16B	109.0	C1B—C2B—N1B	118.60 (18)
H16A—C16A—H16B	107.8	C2B—C3B—C4B	118.8 (2)
N1A—C17A—H17A	109.5	C2B—C3B—H3BA	120.6
N1A—C17A—H17B	109.5	C4B—C3B—H3BA	120.6
H17A—C17A—H17B	109.5	C5B—C4B—C3B	121.3 (2)
N1A—C17A—H17C	109.5	C5B—C4B—N2B	118.8 (2)
H17A—C17A—H17C	109.5	C3B—C4B—N2B	119.9 (2)
H17B—C17A—H17C	109.5	C6B—C5B—C4B	119.3 (2)
N1A—C18A—H18A	109.5	C6B—C5B—H5BA	120.4
N1A—C18A—H18B	109.5	C4B—C5B—H5BA	120.4
H18A—C18A—H18B	109.5	C5B—C6B—C1B	124.4 (2)
N1A—C18A—H18C	109.5	C5B—C6B—N3B	116.5 (2)
H18A—C18A—H18C	109.5	C1B—C6B—N3B	119.1 (2)
H18B—C18A—H18C	109.5		
			
C1C—O1A—C1A—C9A	−68.1 (5)	C5C—C6C—C7C—C2C	0.0
C15A—O1A—C1A—C9A	173.7 (3)	C5C—C6C—C7C—C8C	178.4 (6)
C1C—O1A—C1A—C2A	58.2 (5)	C3C—C2C—C7C—C6C	0.0
C15A—O1A—C1A—C2A	−60.0 (4)	C1C—C2C—C7C—C6C	−179.4 (6)
O1A—C1A—C2A—C3A	−33.7 (4)	C3C—C2C—C7C—C8C	−178.4 (6)
C9A—C1A—C2A—C3A	90.8 (4)	C1C—C2C—C7C—C8C	2.2 (8)
O1A—C1A—C2A—C7A	150.0 (3)	C6C—C7C—C8C—C8C^i^	−19.3 (14)
C9A—C1A—C2A—C7A	−85.5 (4)	C2C—C7C—C8C—C8C^i^	159.1 (10)
C7A—C2A—C3A—C4A	0.0	O1A—C1C—C9C—C10C	−113.0 (6)
C1A—C2A—C3A—C4A	−176.3 (4)	C2C—C1C—C9C—C10C	113.7 (6)
C2A—C3A—C4A—C5A	0.0	O1A—C1C—C9C—C14C	68.7 (9)
C3A—C4A—C5A—C6A	0.0	C2C—C1C—C9C—C14C	−64.6 (8)
C4A—C5A—C6A—C7A	0.0	C14C—C9C—C10C—C11C	0.0
C5A—C6A—C7A—C2A	0.0	C1C—C9C—C10C—C11C	−178.3 (8)
C5A—C6A—C7A—C8A	−178.8 (3)	C9C—C10C—C11C—C12C	0.0
C3A—C2A—C7A—C6A	0.0	C10C—C11C—C12C—C13C	0.0
C1A—C2A—C7A—C6A	176.2 (4)	C11C—C12C—C13C—C14C	0.0
C3A—C2A—C7A—C8A	178.7 (3)	C12C—C13C—C14C—C9C	0.0
C1A—C2A—C7A—C8A	−5.0 (4)	C10C—C9C—C14C—C13C	0.0
O1A—C1A—C9A—C10A	−113.1 (3)	C1C—C9C—C14C—C13C	178.2 (9)
C2A—C1A—C9A—C10A	119.2 (4)	O1B—C1B—C2B—C3B	177.3 (2)
O1A—C1A—C9A—C14A	66.9 (4)	C6B—C1B—C2B—C3B	−0.8 (3)
C2A—C1A—C9A—C14A	−60.8 (4)	O1B—C1B—C2B—N1B	−1.3 (3)
C14A—C9A—C10A—C11A	0.0	C6B—C1B—C2B—N1B	−179.36 (19)
C1A—C9A—C10A—C11A	180.0 (3)	O3B—N1B—C2B—C3B	−29.4 (3)
C9A—C10A—C11A—C12A	0.0	O2B—N1B—C2B—C3B	148.4 (2)
C10A—C11A—C12A—C13A	0.0	O3B—N1B—C2B—C1B	149.3 (2)
C11A—C12A—C13A—C14A	0.0	O2B—N1B—C2B—C1B	−32.9 (3)
C12A—C13A—C14A—C9A	0.0	C1B—C2B—C3B—C4B	1.5 (3)
C10A—C9A—C14A—C13A	0.0	N1B—C2B—C3B—C4B	−179.84 (19)
C1A—C9A—C14A—C13A	−180.0 (3)	C2B—C3B—C4B—C5B	−0.6 (3)
C1C—O1A—C15A—C16A	−152.5 (5)	C2B—C3B—C4B—N2B	179.8 (2)
C1A—O1A—C15A—C16A	−92.8 (3)	O5B—N2B—C4B—C5B	−7.2 (4)
C17A—N1A—C16A—C15A	161.73 (19)	O4B—N2B—C4B—C5B	174.2 (2)
C18A—N1A—C16A—C15A	−74.8 (2)	O5B—N2B—C4B—C3B	172.5 (2)
O1A—C15A—C16A—N1A	−65.3 (3)	O4B—N2B—C4B—C3B	−6.2 (3)
C1A—O1A—C1C—C9C	−53.1 (6)	C3B—C4B—C5B—C6B	−1.1 (3)
C15A—O1A—C1C—C9C	43.5 (9)	N2B—C4B—C5B—C6B	178.5 (2)
C1A—O1A—C1C—C2C	77.7 (6)	C4B—C5B—C6B—C1B	1.9 (4)
C15A—O1A—C1C—C2C	174.4 (4)	C4B—C5B—C6B—N3B	−178.1 (2)
O1A—C1C—C2C—C3C	−43.8 (7)	O1B—C1B—C6B—C5B	−179.0 (2)
C9C—C1C—C2C—C3C	90.7 (6)	C2B—C1B—C6B—C5B	−1.0 (3)
O1A—C1C—C2C—C7C	135.6 (5)	O1B—C1B—C6B—N3B	0.9 (3)
C9C—C1C—C2C—C7C	−89.8 (7)	C2B—C1B—C6B—N3B	178.98 (19)
C7C—C2C—C3C—C4C	0.0	O6B—N3B—C6B—C5B	−21.7 (3)
C1C—C2C—C3C—C4C	179.5 (6)	O7B—N3B—C6B—C5B	156.2 (2)
C2C—C3C—C4C—C5C	0.0	O6B—N3B—C6B—C1B	158.3 (2)
C3C—C4C—C5C—C6C	0.0	O7B—N3B—C6B—C1B	−23.8 (3)
C4C—C5C—C6C—C7C	0.0		

Symmetry codes: (i) −*x*+2, −*y*+2, −*z*+1.

### Hydrogen-bond geometry (Å, °)

**Table:**

Cg2 and Cg3 are the centroids of the C9A–C7A and C2C–C7C rings, respectively.

**Table:**

*D*—H···*A*	*D*—H	H···*A*	*D*···*A*	*D*—H···*A*
N1A—H1AB···O1B	0.93	1.85	2.661 (2)	144.
N1A—H1AB···O7B	0.93	2.36	3.031 (3)	129.
C4A—H4AA···O4B^ii^	0.95	2.46	3.346 (4)	155.
C16A—H16A···O3B^iii^	0.99	2.57	3.519 (3)	160.
C17A—H17A···O2B^iii^	0.98	2.57	3.470 (4)	153.
C18A—H18A···O6B^iv^	0.98	2.41	3.167 (3)	133.
C18A—H18C···O4B^v^	0.98	2.36	3.317 (3)	166.
C8C—H8CB···O6B	0.96	2.48	3.239 (9)	136.
C6A—H6AA···Cg2^vi^	0.93	2.88	3.643 (2)	138.
C6A—H6AA···Cg3^vi^	0.93	3.00	3.836 (4)	148.
C12C—H12B···Cg2^vi^	0.93	2.62	3.492 (4)	153.
C12C—H12B···Cg3^vi^	0.93	2.83	3.704 (4)	153.

Symmetry codes: (ii) *x*, *y*, *z*−1; (iii) −*x*+1, −*y*+1, −*z*+2; (iv) −*x*+2, −*y*+1, −*z*+1; (v) −*x*+2, −*y*+1, −*z*+2; (vi) −*x*+1, −*y*+2, −*z*+1.

### Table 2 Cg···Cg π-stacking interactions (Å) Cg2, Cg3 and Cg5 are the centroids of rings C9A–C14A, C2C–C7C and C1B–C6B. Symmetry code: (i) 1-x, 2-y, 1-z

**Table:**

	*Cg*I···*Cg*J (Å)	CgI···Perp (Å)	CgJ···Perp (Å)
Cg2···Cg5^i^	3.677 (2)	-3.616 (2)	-3.6243 (9)
Cg3···Cg5^i^	3.515 (3)	-3.374 (3)	3.3844 (9)
